# Early and Mid-Term Outcomes of Transcatheter Aortic Valve Implantation versus Surgical Aortic Valve Replacement: Updated Systematic Review and Meta-Analysis

**DOI:** 10.3390/jcdd10040157

**Published:** 2023-04-05

**Authors:** Tsahi T. Lerman, Amos Levi, Yeela Talmor-Barkan, Ran Kornowski

**Affiliations:** 1 Department of Internal Medicine F-Recanati, Beilinson Hospital, Rabin Medical Center, Petah Tikva 4941492, Israel; 2 Department of Cardiology, Rabin Medical Center, Petah Tikva 4941492, Israel; 3 The Faculty of Medicine, Tel Aviv University, Tel Aviv 6997801, Israel

**Keywords:** TAVI, SAVR, transcatheter aortic valve implantation, surgical aortic valve replacement

## Abstract

(1) Background: The use of transcatheter aortic valve implantation (TAVI) for the treatment of severe symptomatic aortic stenosis is expanding significantly. We aimed to perform a meta-analysis comparing the safety and efficacy of TAVI versus surgical aortic valve replacement (SAVR) during the early and mid-term follow-up period. (2) Methods: We conducted a meta-analysis of randomized controlled trials (RCTs) comparing 1- to 2-year outcomes between TAVI and SAVR. The study protocol was preregistered in PROSPERO and the results were reported according to PRISMA guidelines. (3) Results: The pooled analysis included data from eight RCTs totaling 8780 patients. TAVI was associated with a lower risk of all-cause mortality or disabling stroke (OR 0.87, 95%CI 0.77–0.99), significant bleeding (OR 0.38, 95%CI 0.25–0.59), acute kidney injury (AKI; OR 0.53, 95%CI 0.40–0.69) and atrial fibrillation (OR 0.28, 95%CI 0.19–0.43). SAVR was associated with a lower risk of major vascular complication (MVC; OR 1.99, 95%CI 1.29–3.07) as well as permanent pacemaker implantation (PPI; OR 2.28, 95%CI 1.45–3.57). (3) Conclusions: TAVI compared with SAVR during early and mid-term follow-up was associated with a lower risk of all-cause mortality or disabling stroke, significant bleeding, AKI and atrial fibrillation; however, it was associated with a higher risk of MVC and PPI.

## 1. Introduction

Since its introduction more than 20 years ago by Dr. Alain Cribier, transcatheter aortic valve implantation (TAVI) has become the intervention of choice for severe symptomatic aortic valve stenosis in high-risk and elderly patients [1,2]. Continuous research and development of the valve systems as well as imaging techniques along with growing operator experience has led to major improvements in the safety and efficacy of the procedure [3,4,5,6]. This has led to an expansion of the procedure to a broader population, such as patients with a lower surgical risk and with a longer life expectancy [3,6,7,8,9,10,11].

A recently published comprehensive meta-analysis compared the safety and efficacy of TAVI versus SAVR during 1 year of follow-up as well as for the longest data available [12]. In this meta-analysis, among lower risk patients treated by TAVI there was an early mortality reduction as well as a lower risk of the composite outcome of death or disabling stroke, however, no differences were found during the longer follow-up and among the higher-risk patients. In addition, during the short-term follow up, TAVI was associated with a lower risk of major bleeding and acute kidney injury (AKI) and a higher risk of pacemaker implantation. Nevertheless, several important clinical questions remain unresolved: (1) comparative assessment of clinically important secondary outcomes beyond a one-year follow-up, (2) differentiation of the outcomes between self-expanding and balloon-expandable TAVI valves, (3) examination of the impact of surgical risk on the effect size of clinical outcomes using advanced techniques such as meta-regression analysis.

We therefore aimed to conduct an updated systematic review and meta-analysis comparing early and mid-term (1–2 years) safety and efficacy outcomes from all RCTs comparing TAVI and SAVR, using the subgroup analysis according to the valve system types and a meta-regression analysis examining the effect of surgical risk on the primary outcome.

## 2. Methods

The study protocol was written by T.L. and A.L. We conducted a comprehensive search to identify studies in Pubmed, Embase, and Cochrane Central Register of Controlled Trials (CENTRAL), up to December 2022, using a combination of keywords and MeSH terms for: transcatheter aortic valve replacement, transcatheter aortic valve implantation and surgical aortic valve implantation. References of all the included trials and reviews identified were scanned for additional studies. All the titles and abstracts were screened, and those thought to possibly meet the inclusion criteria were screened for eligibility using the full text. The primary outcome was all-cause mortality. The secondary outcomes were cardiovascular mortality, stroke, disabling stroke, composite outcome of all-cause mortality or disabling stroke, acute kidney injury (AKI), major/life-threating or disabling bleeding (significant bleeding), myocardial infarction (MI), permanent pacemaker implantation (PPI), major vascular complications (MVC) and atrial fibrillation. We included studies with a follow-up period between 1 and 2 years. The longest follow-up available data were used in the pooled analysis. Additional data regarding the longest follow-up available regarding the primary outcome were extracted as well.

Two reviewers (T.L., A.L.) independently extracted the data and resolved conflicts by discussion.

Two authors (T.L., A.L.) assessed the risk of bias. Cochrane’s handbook tool was used to assess the studies [13]. A funnel plot was used to assess publication bias. A systematic review and meta-analysis were performed in compliance with the Cochrane Collaboration and Preferred Reporting Items for Systematic Reviews and Meta-Analyses Statement [13]. Meta-analysis was performed using the Review Manager (RevMan, Version 5.4. Copenhagen, Denmark: The Nordic Cochrane Centre, The Cochrane Collaboration, 2020). Meta-regression analysis was performed using Comprehensive Meta-Analysis (Version 4.0. Englewood, NJ, USA: Biostat Inc. 2022). A subgroup analysis graph was created in RSTUDIO Version 2022.02.2 (Package-forestplot).

Heterogeneity between the included trials was assessed using the chi-squared test for heterogeneity and the I^2^ measure of inconsistency. We used a fixed effect model with the Mantel–Haenszel method for pooling trial results throughout the review unless statistically significant heterogeneity was found (*p* < 0.10 or I^2^ > 50%), in which case we chose a random-effects model and used the inverse variance method. Dichotomous data were analysed by calculating the odds ratio (OR) for each trial with 95% confidence interval (CI). Reported values are 2 tailed, and hypothesis-testing results were considered significant at *p* < 0.05.

A sensitivity analysis was carried out examining the effect of the exclusion of each study on the pooled results (“leave-one-out” analysis). A subgroup analysis according to the TAVI valve system used was done as well. A meta-regression examining the association between patient surgical risk according to STS score on the effect size of the primary outcome was carried out as well.

The study was pre-registered in PROSPERO; ID CRD42023391959.

## 3. Results

**Figure 1:**
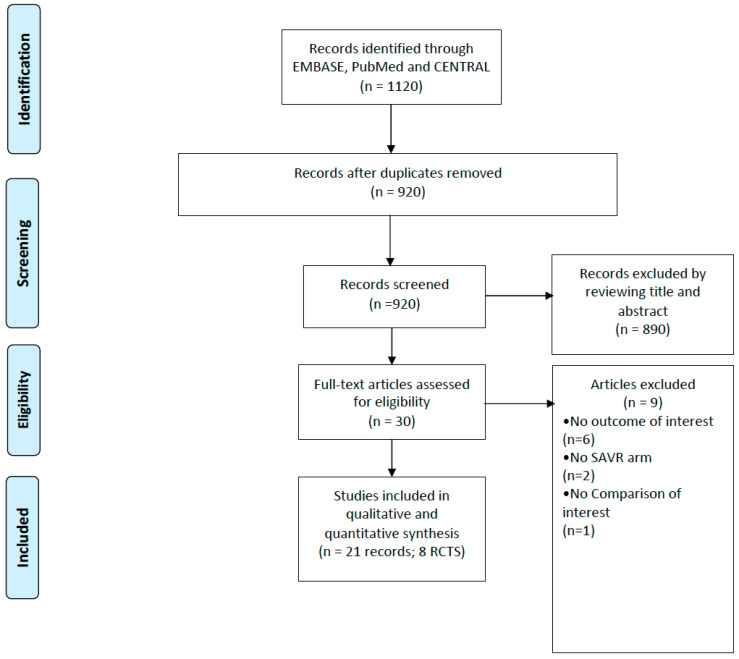
Study selection process for inclusion in the meta-analysis (PRISMA flow diagram).

A flow chart representing the study selection process is shown in Figure 1. Our initial search yielded 920 citations, of which 30 were judged to be potentially eligible and underwent full text review. Twenty-one publications from eight RCTs (PARTNER 1, PARTNER 2, PARTNER 3, US CoreValve high-risk, NOTION, SURTAVI, Evolut Low risk, UK TAVI) were found to be eligible for inclusion after the full text review [3,4,5,6,7,9,10,14,15,16,17,18,19,20,21,22,23,24,25,26,27]. Overall, our primary outcome meta-analysis included data on 8780 patients: 4455 treated by TAVI and 4325 by SAVR. The characteristics of the studies and patients included in this meta-analysis are shown in Table 1.

### 3.1. All-Cause Mortality

All eight RCTs provided data regarding all-cause mortality. In all the included trials, there were data regarding the outcome at a two-year follow-up period, except for the UK TAVI which only reported one year of follow-up.

**Figure 2:**
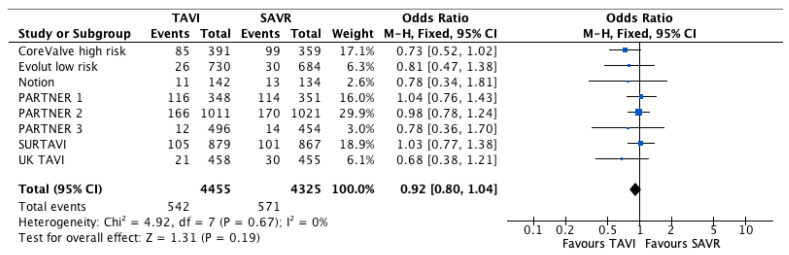
Forest plots for all-cause mortality.

There were 542/4455 (12.2%) deaths in the TAVI group and 571/4325 (13.2%) in the SAVR group. Overall, there was no significant difference between the two groups (OR 0.92 [95% CI; 0.8–1.04], *p* = 0.19, I^2^ = 0%). The forest plot of all-cause mortality is shown in Figure 2. Analysis of the longest data available is presented in Figure S1; the risk for all-cause mortality was higher in the TAVI group for the longest data available (OR 1.19, 95% CI [1.07–1.32], *p* < 0.01, I^2^ = 40%), however, there was a significant heterogeneity between the follow up groups (*p* = 0.02).

In a meta-regression analysis, there was no correlation between the all-cause mortality effect size (log STS score; *p* = 0.45). A meta-regression analysis is shown is Figure S2. The funnel plot is presented in Figure S3.

### 3.2. Secondary Outcomes

TAVI was associated with a lower risk of all-cause mortality or disabling stroke. There were 569/4313 (13.2%) cases in the TAVI group versus 624/4191 (14.9%) in the SAVR group (OR 0.87, 95% CI [0.77–0.99], *p* = 0.03, I^2^ = 0). Additionally, TAVI was associated with a lower risk of significant bleeding. There were 454/3434 (13.2%) cases in the TAVI group and 974/3324 (29.3%) in the SAVR group (OR 0.38, 95% CI [0.25–0.59], *p* < 0.01, I^2^ = 90%). The risk of AKI was lower as well: 90/2942 (3.1%) in the TAVI group versus 161/2920 (5.5%) in the SAVR group (OR 0.53, 95% CI [0.40–0.69], *p* < 0.01, I^2^ = 46%). The risk of new onset atrial fibrillation was lower as well among the TAVI group: 399/3255 (12.3%) in the TAVI group versus 1032/3116 (33.1%) in the SAVR group (OR 0.28, 95% CI [0.19–0.43], *p* < 0.01, I^2^ = 89%).

There was a lower risk for cardiovascular mortality in the TAVI group that did not reach statistical significance (OR 0.92,95% CI [0.79–1.08], *p* = 0.29, I^2^ = 0).

SAVR was associated with a lower risk of MVC: 185/2976 (6.2%) in the TAVI group versus 106/2869 (3.7%) in the SAVR group (OR 1.99, 95% CI [1.29–3.07], *p* < 0.01, I^2^ = 59%). In addition, the risk of PPI was lower in the SAVR group: 551/3229 (17.1%) in the TAVI group versus 279/3110 (9%) in the SAVR group (OR 2.28, 95% CI [1.45–3.57], *p* < 0.01, I^2^ = 86%).

**Figure 3:**
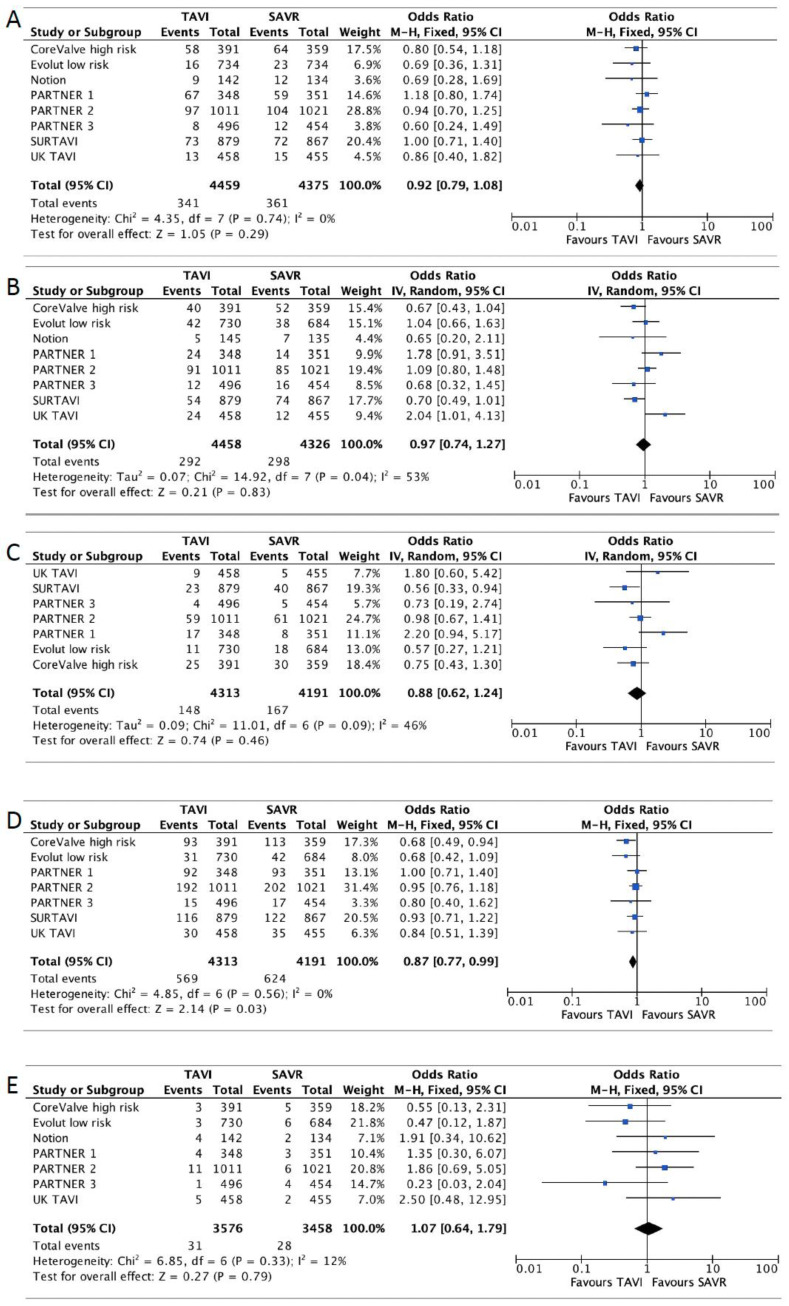
Forest plots for cardiovascular mortality (**A**), stroke (**B**), disabling stroke (**C**), all-cause mortality or disabling stroke (**D**), endocarditis (**E**).

**Figure 4:**
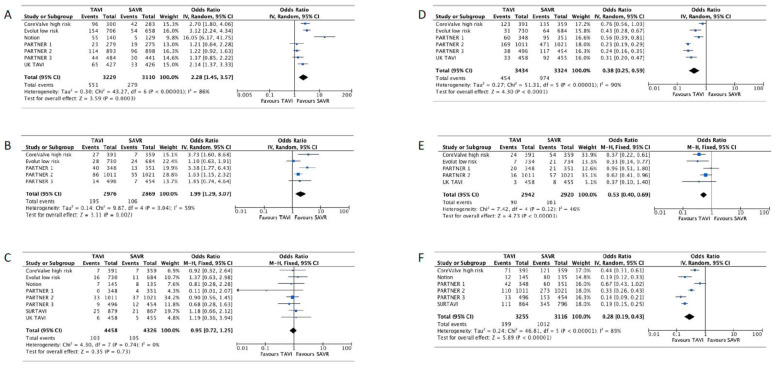
Forest plot for permanent pacemaker implantation (**A**), major vascular complication (**B**), myocardial infarction (**C**), significant bleeding (**D**), acute kidney injury (**E**), atrial fibrillation (**F**).

There was no statistically significant difference regarding the risk of stroke (OR 0.97, 95% CI [0.74–1.27], *p* = 0.83, I^2^ = 53%), disabling stroke (OR 0.88, 95% CI [0.62–1.24], *p* = 46), MI (OR 0.95, 95% CI [0.72–1.25], *p* = 0.73, I^2^ = 0), or endocarditis (OR 1.07, 95% CI [0.64–1.79], *p* = 79, I^2^ = 12%). Secondary outcomes forest plots are shown in Figure 3 and Figure 4. The results are summarized in Table 2.

### 3.3. Sensitivity Analysis

Following the exclusion of the EVOLUT low risk as well as UC CoreValve high risk, the composite outcome of all-cause mortality of disabling stroke loss its statistical significance.

No other study exclusion yielded a change in the effect size direction or in its statistical significance.

### 3.4. Subgroup Analysis

**Figure 5:**
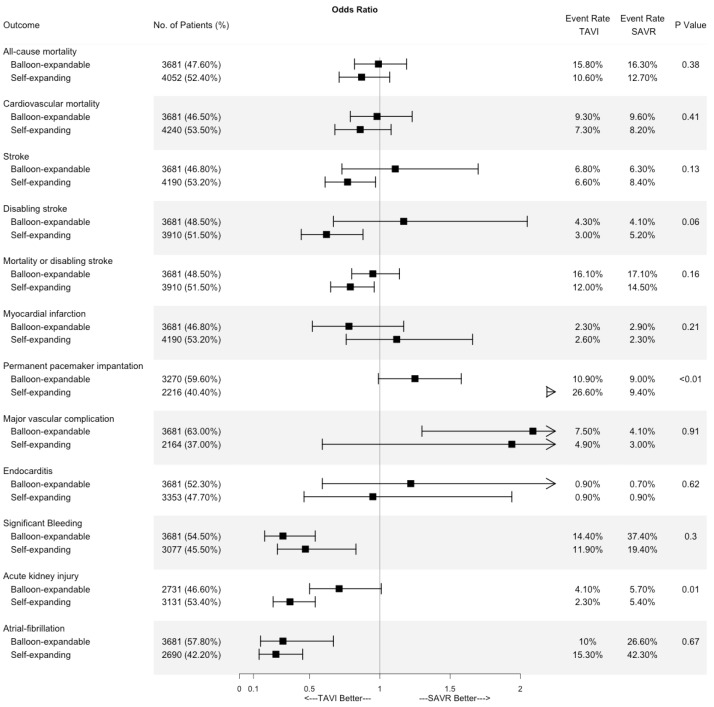
Subgroup analysis for main outcomes according to TAVI valve system. Range represents Odds Ratio and 95% confidence interval.

All the RCTs except the UK TAVI included the TAVI arm with the homogenous valve system used with regards to the balloon-expandable or self-expanding valve. Unfortunately, the UK TAVI study used heterogenous valve systems and did not provide a subgroup or sensitivity analysis comparing the valve systems and, therefore, sub-group analysis could not be conducted using this study. Consequently, it was excluded from the subgroup meta-analysis. As mentioned above, its exclusion did not result in a shift in the effect size direction of the statistical significance of any outcome of interest. There were significant interactions between the valve type and PPI (*p* < 0.01) as well as AKI (*p* = 0.01). Subgroup analysis is presented in Figure 5. Forest plots for PPI and AKI are presented in Figure S4.

## 4. Discussion

We have conducted a systematic review and meta-analysis which included all the RCTs comparing the safety and efficacy between TAVI and SAVR for the early and mid-term follow up period. At the two-year follow-up, we found no significant difference in the all-cause mortality between the TAVI and SAVR groups. TAVI was associated with a lower risk of all-cause mortality or disabling stroke, significant bleeding, acute kidney injury (AKI) and atrial fibrillation compared to SAVR. On the other hand, SAVR was associated with a lower risk of MVC and PPI compared to TAVI. There was no significant difference in the risk of stroke, disabling stroke, MI, or endocarditis between the two groups.

In the latest meta-analysis published by Ahmad et al., all-cause mortality was reported for 1 year of follow-up and the longest follow-up available separately [12]. Both comparisons did not find a statistically significant difference between the two groups, however, a clear trend was demonstrated for lower mortality during short-term follow-up. Since studies with longer follow-up were pooled ranging from 2 to 8 years of follow-up no comparison or conclusion can be made regarding the mid-term outcome. Interestingly, a previous meta-analysis published in 2019 focusing on mid-term outcomes found a lower rate of all-cause mortality among the TAVI group [8]. This discrepancy can be attributed to several factors. Firstly, the current study as well as the meta-analysis by Ahmad et al. included two studies with a longer follow-up period compared to the previous study (PARTNER 3 and Evolut low risk), in addition to the UK TAVI that was not published at the time of the previous meta-analysis [9,15,24]. Furthermore, we employed a statistical method based on the event rate and calculated odds ratio instead of the hazard ratio, which further contributed to the difference. An important finding in our study is that, upon conducting a meta-regression analysis, no correlation was identified between the surgical risk of the patients and the effect size of the primary outcome at the study level. In the previous meta-analyses, subgroup analysis was used according to surgical risk, but in our opinion, the use of a meta-regression technique is more appropriate in this case [8,12].

Our analysis found that the risk of all-cause mortality was higher in the TAVI group when looking at the longest available follow-up period. However, the data showed a significant discrepancy between the follow-up periods, making it unclear whether this difference is significant. In studies with a 5-year follow-up, a survival advantage was found in the SAVR group; this contrasts with short-term studies in which there was a trend towards an advantage for the TAVI group. This difference is probably explained by the fact that early studies which reported long-term follow-up data were characterized by less experience of the operators, older patients with a higher rate of comorbidities and a higher surgical risk. In addition, only one study examined 8-year outcomes. Therefore, further research with a longer follow-up period is needed to fully understand and clarify this finding.

Another key difference between the current study and the recent meta-analysis by Ahmad et al. compared to the previous meta-analyses by Sinotis et al. is that we did not observe a statistically significant difference in stroke rates [8,12]. The reasons for these dissimilarities were similar to the previously mentioned factors. Additionally, it is worth noting that the stroke rate in the UK TAVI study TAVI group was double that of the SAVR group stroke rate [15]. Although its exclusion did not change the statistical significance of the analysis, there was a clear trend towards an advantage for the TAVI group in the analysis that excluded the UK TAVI data. Importantly, TAVI was associated with a lower risk of all-cause mortality or disabling stroke. Given that neurological complications are a major concern, especially in the era of performing the procedure in declining patient age and risk, this finding should be a key factor in the decision-making process for both the heart team and in the discussion between cardiologists and patients when evaluating the options available. The non-significant trend of improved mortality and a decrease in the stroke/death composite outcome may be attributed to the differences observed in secondary outcomes. First, we found a notably lower rate of significant bleeding among the TAVI group. In addition, we also found a reduced incidence of AKI in the TAVI group, which can be attributed to a reduced rate of bleeding [28,29]. This finding is in keeping with previous research, which has identified AKI as a predictor of mortality and poor prognosis [30]. In addition, the risk of atrial fibrillation was lower among the TAVI group. The benefits of TAVI regarding the secondary outcomes of AKI, bleeding and atrial fibrillation have been previously supported in meta-analyses [8,12].

Our study revealed a notable correlation between the type of TAVI valve used and the risk of AKI. However, a previous meta-analysis conducted by our group did not identify any significant difference in the AKI risk between the latest generation two-valve systems [31]. Further research is required to elucidate this issue.

The current study highlights two significant safety disadvantages of TAVI, which are well established [8,12]. The first is a higher rate of MVC. The second finding is the higher rate of PPI. This finding is consistent across all studies included and is particularly pronounced with the use of self-expanding valves. A recent meta-analysis by our group also recognized this issue in the latest generation self-expanding valves, emphasizing the need for improved safety in the design of future valves [31].

Our meta-analysis has several strengths that make it a valuable contribution to the field. Primarily, it is the most comprehensive and up-to-date meta-analysis published that compares early and mid-term “hard outcomes” between TAVI and SAVR, therefore, providing important insights for heart teams to consider when making treatment decisions. In addition, the pooled analysis only included data from RCTs, which provides a high level of quality and reliability for the analysis. The exclusion of observational study data further enhances the strength of our analysis by minimizing the potential for bias and increasing the generalizability of our findings. Moreover, we used advanced statistical methods, such as meta-regression analysis, to examine the relationship between surgical risk and the main outcome. Finally, we conducted a subgroup analysis by valve type to control for possible confounding factors and increase the validity of the study findings. There are several limitations to this study that should be noted. One limitation is that some of the studies included in our analysis used older generation valves, which may not accurately reflect the outcomes of modern TAVI procedures. Another limitation is that we did not have access to individual patient data and our analysis was based on aggregate data. This means that the results should be interpreted with caution and may not be generalizable to all patients. Additionally, some of the analyses revealed significant heterogeneity between the studies, which could indicate that the results may not be directly comparable. Lastly, our analysis examined only early and mid-term outcomes up to 2 years of follow-up. However, we believe that at present, there is insufficient long-term data to conduct a comprehensive “hard-outcome” meta-analysis and further studies with longer-term data are necessary to establish a definitive conclusion within this time frame.

In conclusion, our analysis found no significant difference in the all-cause mortality between TAVI and SAVR. TAVI was associated with a lower risk of all-cause mortality or disabling stroke, as well as a lower risk of significant bleeding, AKI and atrial fibrillation. However, it was also associated with a higher risk of major vascular complications and the need for permanent pacemaker implantation. Our analysis provides insights that should be considered in the decision-making process for both the heart team and in the shared decision making between cardiologists and patients.

## Figures and Tables

**Table 1:** Characteristics of studies and patients included in the meta-analysis.

Study	Year	Sample Size	Longest Follow Up	TAVI Valve	STS (mean ± S.D)	EuroScore (mean ± S.D)	Age (mean ± S.D)	Male	Risk of Bias
PARTNER 1	2011	TAVI 348	5 years	Sapien (balloon-expandable)	11.8 ± 3.3	29.3 ± 16.5 *	83.6 ± 6.8	58%	Low
		SAVR 351			11.7 ± 3.5	29.2 ± 15.6 *	84.5 ± 6.4	57%	
PARTNER 2	2016	TAVI 1011	5 years	Sapien XT (balloon-expandable)	5.8 ± 2.1	-	81.5 ± 6.7	54%	Low
		SAVR 1021			5.8 ± 1.9	-	81.7 ± 6.7	55%	
PARTNER 3	2019	TAVI 496	2 years	Sapien 3 (balloon-expandable)	1.9 ± 0.7	1.5 ± 1.2	73.3 ± 5.8	68%	Low
		SAVR 454			1.9 ± 0.6	1.5 ± 0.9	73.6 ± 6.1	71%	
CoreValve	2014	TAVI 394	5 years	CoreValve (self-exapnding)	7.3 ± 3.0	17.6 ± 13.0 *	83.2 ± 7.1	54%	Some concerns
		SAVR 401			7.5 ± 3.2	18.4 ± 12.8 *	83.5 ± 6.3	47%	
NOTION	2015	TAVI 145	8 years	CoreValve (self-exapnding)	2.9 ± 1.6	1.9 ± 1.2	79.2 ± 4.9	54%	Low
		SAVR 135			3.1 ±1.7	2.0 ± 1.3	79.0 ± 4.7	53%	
SURTAVI	2017	TAVI 879	5 years	CoreValve/Evolut R (self-exapnding)	4.4 ± 1.5	11.9 ± 7.6 *	79.9 ± 6.2	58%	Low
		SAVR 867			4.5 ± 1.6	11.6 ± 8.0 *	79.8 ± 6.0	56%	
Evolut	2019	TAVI 734	2 years	CoreValve/Evolut R/Pro (self-exapnding)	1.9 ± 0.7	-	74.0 ± 5.9	64%	Low
		SAVR 734			1.9 ± 0.7	-	73.8 ± 6.0	66%	
UK TAVI	2022	TAVI 458	1 year	Various models (12 types)	2.6	2	81	54%	Low
		SAVR 455			2.7	2	81	53%	

* EuroScore, in other studies EuroScore II is reported. Year refers to first study published.

**Table 2:** Summary of main analysis.

	TAVI	SAVR	OR [95% CI]
All-cause mortality	542/4455 (12.2%)	571/4325 (13.2%)	0.92 [0.80, 1.04]
CV mortality	341/4459 (7.6%)	361/4375 (8.3%)	0.92 [0.79, 1.08]
Stroke	292/4458 (6.6%)	298/4326 (6.9%)	0.97 [0.74, 1.27]
Disabling stroke	148/4313 (3.4%)	167/4191 (4%)	0.88 [0.62, 1.24]
All-cause mortality or disabling stroke	569/4313 (13.2%)	624/4191 (14.9%)	0.87 [0.77, 0.99]
MI	103/4458 (2.3%)	105/3426 (3.1%)	0.95 [0.72, 1.25]
PPI	551/3229 (17.1%)	279/3110 (9%)	2.28 [1.45, 3.57]
MVC	185/2976 (6.2%)	106/2869 (3.7%)	1.99 [1.29, 3.07]
Endocarditis	31/3476 (0.9%)	28/3458 (0.8%)	1.07 [0.64, 1.79]
Significant bleeding	454/3434 (13.2%)	974/3324 (29.3%)	0.38 [0.25, 0.59]
AKI	90/2942 (3.1%)	161/2920 (5.5%)	0.53 [0.40, 0.69]
Atrial Fibrillation	399/3255 (12.3%)	1032/3116 (33.1%)	0.28 [0.19, 0.43]

AKI—Acute kidney injury; CI—Confidence interval; CV—Cardiovascular; MI—Myocardial infarction; MVC—Major vascular complication; OR—Odds ratio; PPI—Permanent pacemaker implantation; TAVI—Transcatheter aortic valve implantation; SAVR—Surgical aortic valve replacement.

## Data Availability

No new data were created or analyzed in this study. Data sharing is not applicable to this article.

## Supplementary Materials

The following supporting information can be downloaded at: https://www.mdpi.com/article/10.3390/jcdd10040157/s1. Figure S1. Longest follow-up available for all-cause mortality; Figure S2. Meta regression analysis of all-cause mortality effect size and surgical risk (STS); Figure S3. Funnel plot for all-cause mortality; Figure S4 Subgroup analysis for Permanent pacemaker implantation (A) and Acute kidney injury (B) according to TAVI valve system.
