# Supplementary material for: Early and Mid-Term Outcomes of Transcatheter Aortic Valve Implantation versus Surgical Aortic Valve Replacement: Updated Systematic Review and Meta-Analysis
Source: J Cardiovasc Dev Dis. 2023 Apr 5;10(4):157. doi: 10.3390/jcdd10040157 (PMC10146134; doi:10.3390/jcdd10040157)

Supplemental Material

Intended for publication as an online data supplement

Figure S1. Longest follow-up available for all-cause mortality.

Study or Subgroup	TAVI Events	TAVI Total	SAVR Events	SAVR Total	Weight	Odds Ratio M-H, Fixed, 95% CI
**3.1.1 1–2 years**						
Evolut low risk	26	730	30	684	4.8%	0.81 [0.47, 1.38]
PARTNER 3	12	496	14	454	2.3%	0.78 [0.36, 1.70]
UK TAVI	21	458	30	455	4.6%	0.68 [0.38, 1.21]
**Subtotal (95% CI)**		**1684**		**1593**	**11.7%**	**0.75 [0.53, 1.07]**
Total events	59		74			
Heterogeneity: $Chi^2 = 0.19$, df = 2 (P = 0.91); $I^2 = 0\%$						
Test for overall effect: Z = 1.60 (P = 0.11)						
**3.1.2 5 years**						
CoreValve high risk	208	391	184	359	14.4%	1.08 [0.81, 1.44]
PARTNER 1	229	348	198	351	10.8%	1.49 [1.10, 2.02]
PARTNER 2	436	1011	370	1021	33.5%	1.33 [1.12, 1.59]
SURTAVI	243	864	200	796	24.0%	1.17 [0.94, 1.45]
**Subtotal (95% CI)**		**2614**		**2527**	**82.7%**	**1.26 [1.12, 1.42]**
Total events	1116		952			
Heterogeneity: $Chi^2 = 3.10$, df = 3 (P = 0.38); $I^2 = 3\%$						
Test for overall effect: Z = 3.95 (P < 0.0001)						
**3.1.3 8 years**						
Notion	75	145	71	135	5.7%	0.97 [0.60, 1.54]
**Subtotal (95% CI)**		**145**		**135**	**5.7%**	**0.97 [0.60, 1.54]**
Total events	75		71			
Heterogeneity: Not applicable						
Test for overall effect: Z = 0.15 (P = 0.88)						
**Total (95% CI)**		**4443**		**4255**	**100.0%**	**1.19 [1.07, 1.32]**
Total events	1250		1097			
Heterogeneity: $Chi^2 = 11.65$, df = 7 (P = 0.11); $I^2 = 40\%$						
Test for overall effect: Z = 3.13 (P = 0.002)						
Test for subgroup differences: $Chi^2 = 8.39$, df = 2 (P = 0.02), $I^2 = 76.2\%$						

Odds Ratio
M–H, Fixed, 95% CI

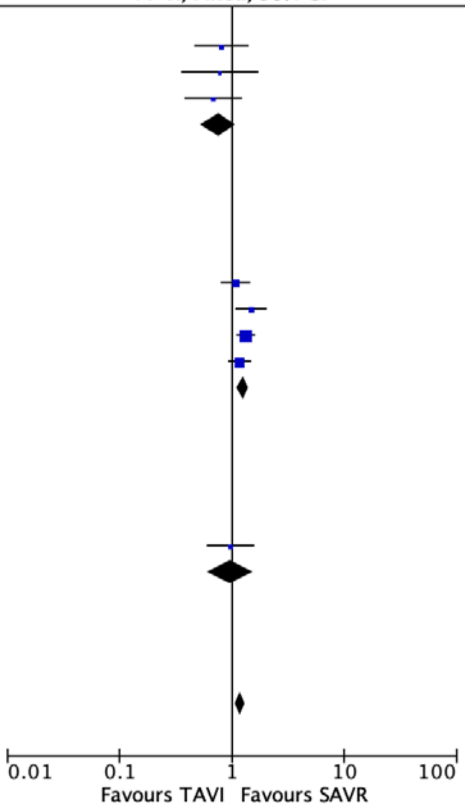

Figure S2. Meta regression analysis of all-cause mortality effect size and surgical risk (STS)

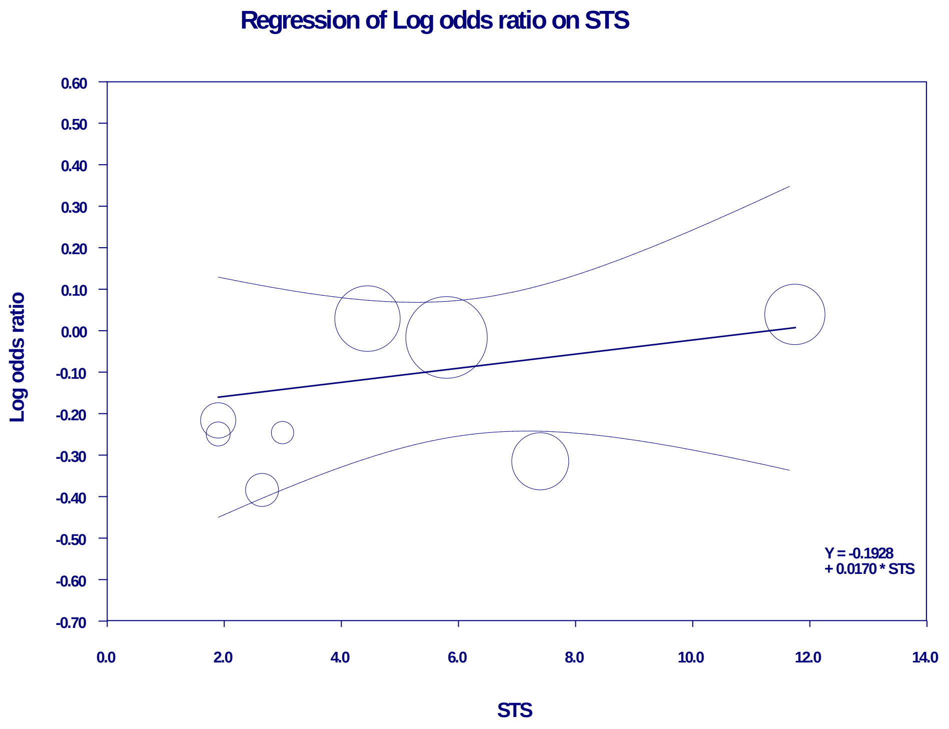

p=0.45

Figure S3. Funnel plot for all-cause mortality.

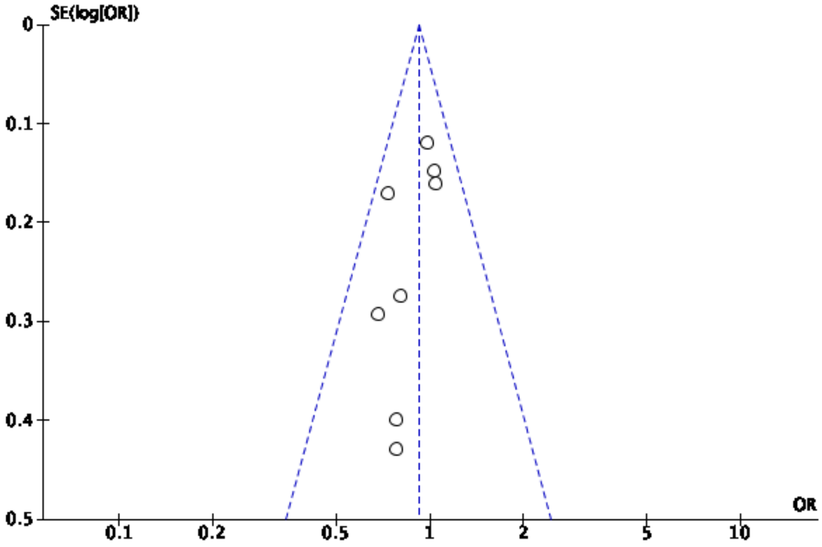

Figure S4 Subgroup analysis for Permanent pacemaker implantation (A) and Acute kidney injury (B) according to TAVI valve system.

A

Study or Subgroup	TAVI Events	TAVI Total	SAVR Events	SAVR Total	Weight	Odds Ratio IV, Random, 95% CI
**2.7.1 Self-expanding**						
PARTNER 1	23	279	19	275	15.6%	1.21 [0.64, 2.28]
PARTNER 2	114	893	96	898	18.8%	1.22 [0.92, 1.63]
PARTNER 3	44	484	30	441	17.1%	1.37 [0.85, 2.22]
**Subtotal (95% CI)**		**1656**		**1614**	**51.5%**	**1.25 [0.99, 1.58]**
Total events	181		145			
Heterogeneity: Tau² = 0.00; Chi² = 0.17, df = 2 (P = 0.92); I² = 0%						
Test for overall effect: Z = 1.92 (P = 0.06)						
**2.7.2 Balloon-expandable**						
CoreValve high risk	96	300	42	283	17.8%	2.70 [1.80, 4.06]
Evolut low risk	154	706	54	658	18.5%	3.12 [2.24, 4.34]
Notion	55	140	5	129	12.1%	16.05 [6.17, 41.75]
**Subtotal (95% CI)**		**1146**		**1070**	**48.5%**	**4.34 [2.19, 8.59]**
Total events	305		101			
Heterogeneity: Tau² = 0.28; Chi² = 11.55, df = 2 (P = 0.003); I² = 83%						
Test for overall effect: Z = 4.20 (P < 0.0001)						
**Total (95% CI)**		**2802**		**2684**	**100.0%**	**2.33 [1.37, 3.97]**
Total events	486		246			
Heterogeneity: Tau² = 0.37; Chi² = 43.14, df = 5 (P < 0.00001); I² = 88%						
Test for overall effect: Z = 3.11 (P = 0.002)						
Test for subgroup differences: Chi² = 11.36, df = 1 (P = 0.0007), I² = 91.2%						

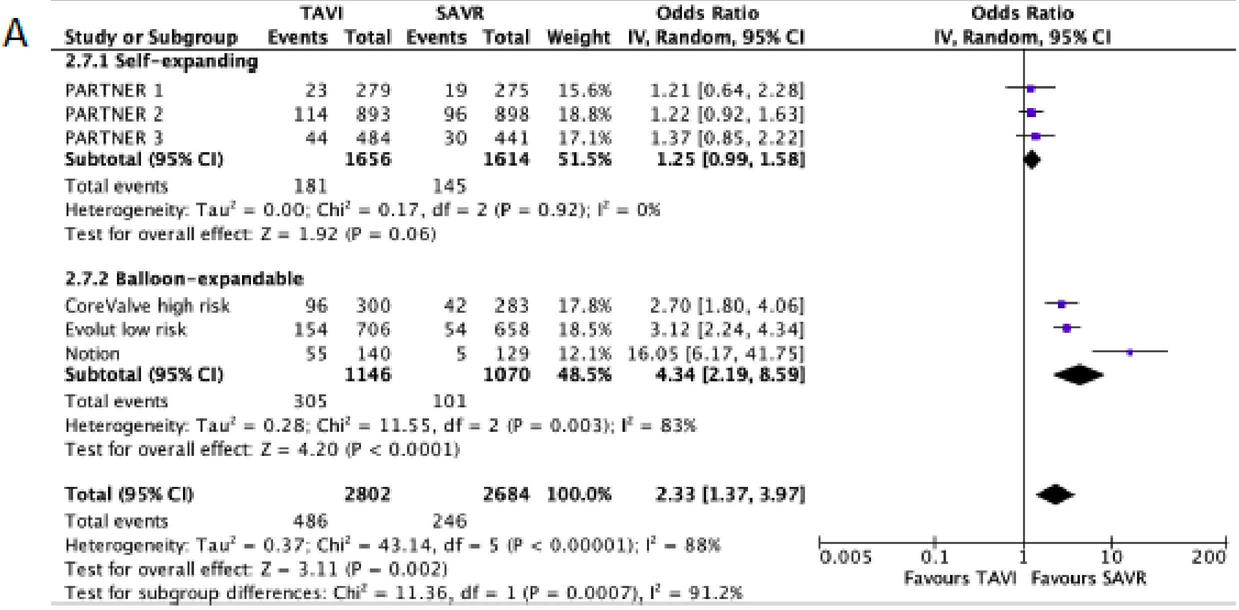

B

Study or Subgroup	TAVI Events	TAVI Total	SAVR Events	SAVR Total	Weight	Odds Ratio M-H, Fixed, 95% CI
**2.11.1 Balloon-expandable**						
PARTNER 1	20	348	21	351	12.6%	0.96 [0.51, 1.80]
PARTNER 2	36	1011	57	1021	35.1%	0.62 [0.41, 0.96]
**Subtotal (95% CI)**		**1359**		**1372**	**47.7%**	**0.71 [0.50, 1.01]**
Total events	56		78			
Heterogeneity: Chi² = 1.21, df = 1 (P = 0.27); I² = 18%						
Test for overall effect: Z = 1.89 (P = 0.06)						
**2.11.2 Self-expanding**						
CoreValve high risk	24	391	54	359	33.9%	0.37 [0.22, 0.61]
Evolut low risk	7	734	21	734	13.3%	0.33 [0.14, 0.77]
UK TAVI	3	458	8	455	5.1%	0.37 [0.10, 1.40]
**Subtotal (95% CI)**		**1583**		**1548**	**52.3%**	**0.36 [0.24, 0.54]**
Total events	34		83			
Heterogeneity: Chi² = 0.06, df = 2 (P = 0.97); I² = 0%						
Test for overall effect: Z = 4.86 (P < 0.00001)						
**Total (95% CI)**		**2942**		**2920**	**100.0%**	**0.53 [0.40, 0.69]**
Total events	90		161			
Heterogeneity: Chi² = 7.42, df = 4 (P = 0.12); I² = 46%						
Test for overall effect: Z = 4.73 (P < 0.00001)						
Test for subgroup differences: Chi² = 6.16, df = 1 (P = 0.01), I² = 83.8%						

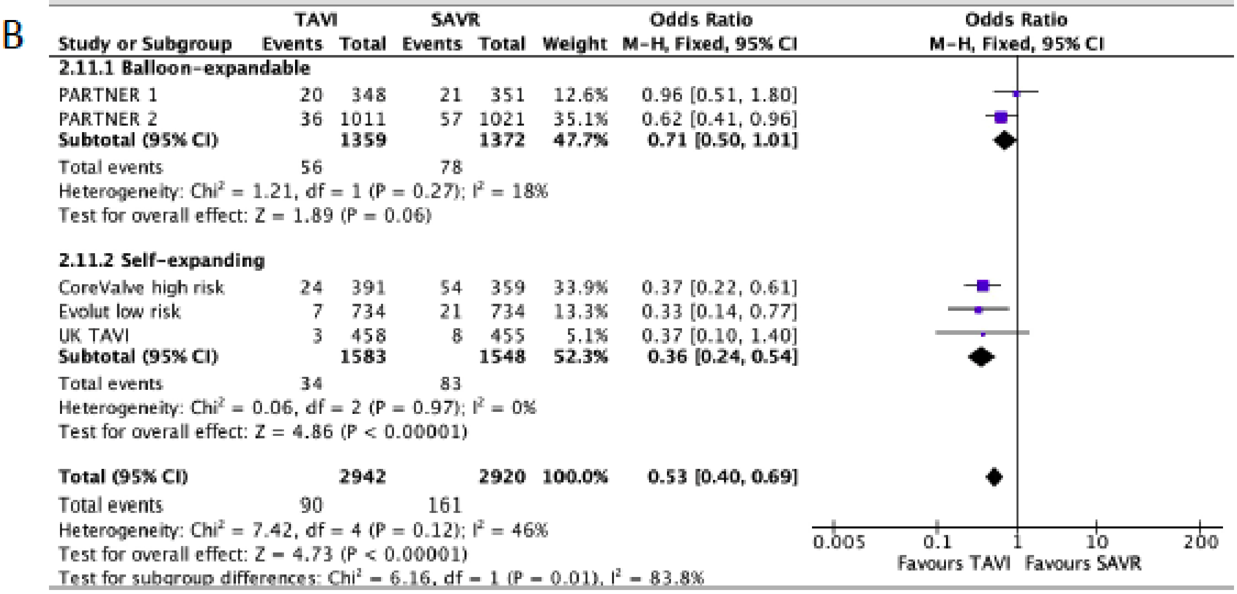

Supplement: Supplementary file 1 [file jcdd-10-00157-s001.zip › jcdd-2311330-supplementary.pdf]
